# Comparative Study on Adaptive Bayesian Optimization for Batch Cooling Crystallization for Slow and Fast Kinetic Regimes

**DOI:** 10.1021/acs.cgd.3c01225

**Published:** 2024-01-29

**Authors:** Thomas Pickles, Chantal Mustoe, Cameron J. Brown, Alastair J. Florence

**Affiliations:** CMAC Future Manufacturing Research Hub, Technology and Innovation Centre, University of Strathclyde, Glasgow G1 1RD, U.K.

## Abstract

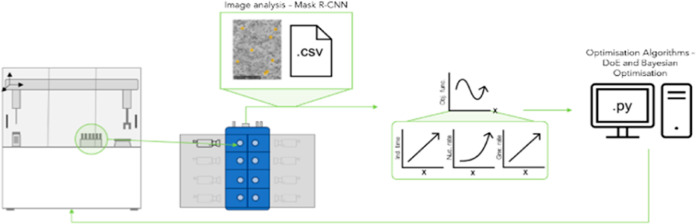

Crystallization kinetic parameter estimation is important for the classification, design, and scale-up of pharmaceutical manufacturing processes. This study investigates the impact of supersaturation and temperature on the induction time, nucleation rate, and growth rate for the compounds lamivudine (slow kinetics) and aspirin (fast kinetics). Adaptive Bayesian optimization (AdBO) has been used to predict experimental conditions that achieve target crystallization kinetic values for each of these parameters of interest. The use of AdBO to guide the choice of the experimental conditions reduced material usage up to 5-fold when compared to a more traditional statistical design of experiments (DoE) approach. The reduction in material usage demonstrates the potential of AdBO to accelerate process development as well as contribute to Net-Zero and green chemistry strategies. Implementation of AdBO can lead to reduced experimental effort and increase efficiency in pharmaceutical crystallization process development. The integration of AdBO into the experimental development workflows for crystallization development and kinetic experiments offers a promising avenue for advancing the field of autonomous data collection exploiting digital technologies and the development of sustainable chemical processes.

## Abstract 2

A comparative study between the design of experiment and Bayesian approaches in designing optimized experiments to both reduce material wastage and drive efficiency in the classification of crystallization kinetics.

## 1 Introduction

Crystallization is a key step in the manufacture of high-quality drug products and serves as a purification step to remove impurities from the crude product.^1^ Understanding and controlling the crystallization of pharmaceuticals allows us to design manufacturing processes that yield the desired particle shape, size, and polymorph without impacting the yield, purity, and quality of the final product. For example, differences in particle shape and size affect downstream processing as well as the effectiveness of dissolution for human absorption.^2^

As particle shape and size are dictated by the kinetics of crystallization, these properties can be controlled by altering key process parameters that dictate supersaturation. Controlling particle shape and size relies on controlling nucleation, growth, and agglomeration rate processes. Primary nucleation, which is the initial formation of new crystals in solution and can be described by induction time, is inherently difficult to control deterministically as it displays a stochastic character.^3^ Secondary nucleation relates to the formation of new nuclei from the attrition of existing crystals and can be controlled by changes in solid loading, particle size, shear rate, mixing, and supersaturation.^4^ A pharmaceutical crystallization process requires nucleation and growth rates that can be maintained within ranges that facilitate robust and controlled operation. Excessive nucleation rates lead to too many fines and/or fouling, and excess particle growth can lead to unfavorable particle shapes, agglomeration, and reduced purification performance with the potential inclusion of impurities. Agglomeration, the aggregation of two or more particles to form larger particles, can change the final product’s physical and chemical properties, and while it can be used to aid in filtering, agglomeration must be controlled to ensure consistency and tablet content uniformity.^5,6^ Conversely, very low nucleation and growth rates can lead to low yields and long, uneconomic processing times.

Various methods have been applied to develop controlled crystallization processes ranging from chemical intuition,^7^ methodical parameter investigation^8^ or machine learning approaches proposed in this work. Design of experiment (DoE)^9^ is a powerful statistical tool used to map the design space, fit models to the data, interpolate between known values and forms a useful component of quality by design (QbD) experimental planning. The use of statistical DoE for process understanding was introduced initially in the agriculture industry^9,10^ and in recent years has seen wide application in biotechnological processing,^11,12^ drug discovery, and medicines manufacture.^13−16^ DoE experimental planning aims to use the least number of experiments to explore the effects of changing a given number of variables across the whole design space. However, even with DoE methods, exploring large numbers of process variables still requires many experiments. For example, a full factorial design exploring five variables with three increments would result in a plan of 243 experiments which, when using expensive active pharmaceutical ingredients (APIs), may not be necessitating using other design methods.

In this work, we explore Bayesian optimization^17^ (BO) as an alternative method to DoE for finding global or local minima or maxima in functions of interest. BO constructs a probabilistic model of the objective function, specifically the difference between target and experimental crystallization kinetic parameters. It employs an acquisition function to iteratively suggest the next point of evaluation, or experiment, to either reduce uncertainty in the model by further exploration or determine the global optimum by exploiting known values. Previous studies^18^ utilizing BO in pharmaceutical crystallization are promising but highlight the need for methods to accommodate the multiple objectives required for suitable process design. For example, Bayesian approaches for optimization of chemical reactions were shown to be efficient, taking a few hours of lab work compared to super modified simplex algorithm (10+ hours per variable) and grid searches (600+ hours per variable).^19^

This work demonstrates the application of algorithms to optimize crystallization process conditions to achieve desired pharmaceutical crystallization kinetic parameters. Methods including DoE and BO can be used to target specific values for pharmaceutical crystallization parameters, and both methods are shown to be significantly more efficient than grid-search approaches. An adaptive (a varying exploration and exploitation model) BO experimental planner showed further improved performance over the DoE and fixed BO methods. The exploration of two API case studies shows the algorithmic approach proposed may be generalizable to other APIs. Implementing improved decision-making algorithms such as these could reduce time and material use and contribute to a greener and more sustainable approach to process development in medicine manufacturing.

## 2 Materials

Lamivudine was purchased from Molekula Ltd. as an off-white powder and aspirin was purchased from Alfa Aesar as a white solid. Both APIs have a purity exceeding 99% and did not undergo further purification. Ethanol and ethyl acetate were purchased from VWR and have purity exceeding 99.97%, so they did not undergo further purification.

## 3 Experimental Methods and Optimization

**Figure 1:**
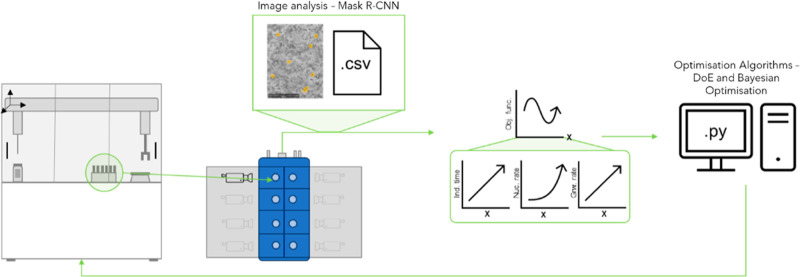
Schematic diagram of the experimental setup and optimization loop consisting of the Zinsser Crissy platform, the Technobis Crystalline, data analysis, and optimization algorithms (left-to-right).

The optimization loop included the following steps: vial dosing, crystallization, analysis of the resulting image data, and further data analysis by an optimization algorithm to recommend the next best round of experiments. A schematic to represent this logical flow is shown in Figure 1.

### 3.1 Vial Dosing, Crystallization, and Image Analysis

Sample preparation for crystallization experiments was carried out using a Zinsser Analytics Crissy platform,^20^ an XYZ robot that doses both powders and liquids. Crystallization experiments were conducted using a Technobis Crystalline platform,^21^ a parallel reactor system that can perform eight separate heating, cooling, and stirring procedures with in-built sample imaging at the 2–7 mL scale.

The experimental procedure was consistent for all experiments and involved the following steps:

1. Heat the solution to a temperature 10 °C below the solvent’s boiling point at a rate of 0.5 °C/min.
2. Maintain the elevated temperature for 10 min to ensure complete dissolution.
3. Cool the solution to the desired isothermal temperature at a rate of −10 °C/min, with no stirring at this stage.
4. Keep the solution at the isothermal temperature for 3 h.
5. Repeat steps 1–4 for a total of five cycles.

The stir rate was fixed at 600 rpm throughout the experiment, except where specified.

Images were captured every 5 s, and an in-house convolutional neural network (CNN) image analysis algorithm^22,23^ was used to extract kinetic parameters. X-ray powder diffraction (XRPD) patterns were collected using a Bruker D8 Discover,^24^ and the data were visualized using DIFFRAC.EVA^25^ and Matplotlib^26^ (Python). Solubility profiles were generated for each API in a solvent selected from previous work.^22,27^

### 3.2 Optimization: Input Parameter Bounds, Target Parameter Objectives, and Approaches

The objective of this optimization problem was to minimize the difference between the experimentally measured values for the kinetic parameters of interest and the associated target values. Table 1 presents the bounds on the input parameters for supersaturation and isothermal experimental temperature and the target objectives for induction time, nucleation rate, and growth rate. As this optimization problem has multiple objectives it can be assumed that the optimum process conditions will sit on a Pareto front^28^ so the objective function value will be used as a quantifier for optimization performance.

**Table 1:** Parameters and Objectives for the Optimization Problem

API	input parameter bounds		target parameter objectives		
	supersaturation	temperature (°C)	induction time (s)	nucleation rate (#/s)	growth rate (μm/s)
lamivudine	2–3	5–50	3600	0.1	0.01
aspirin	1.05–2	5–50	3600	0.1	0.05

The input parameter bounds were varied for each API to accommodate the different sizes of their metastable zone width (MSZW).^22,27^ Lamivudine showed a broad MSZW (>30 °C) and therefore was deemed unlikely to observe any nucleation at low supersaturations within the time constraints of the experiment. Aspirin displayed a narrow MSZW (mean of 16 °C) and thus nucleation was likely to be feasible at low supersaturations (generally below 1.2). As a larger MSZW generally allows higher supersaturations to be achieved before primary nucleation occurs and nucleation is known to dominate growth at high supersaturations,^29^ it was mechanistically assumed that, comparatively, nucleation would dominate for lamivudine crystallization but growth would dominate for aspirin crystallization. Thus, while the nucleation rate target was held constant for both systems, the growth rate target for aspirin was set to 5× the target for lamivudine. Numerical values for rate targets were based on the initial experiments.

#### 3.2.1 Optimization Approach 1: DoE with Surface Minimization

For the DoE optimization, an initial DoE screening of 28 experiments was followed by successive rounds of smaller screens (7 experiments/iteration) centered at the next predicted optimum. We refer to this method as adaptive due to the iterative update of the objective function and the adaptation of this new objective function surface to guide the next round of experiments. This cycle was repeated until the termination of a change in temperature of less than 2 °C and a change in supersaturation of less than 0.02 between the previous and next recommended experiments was achieved.

The initial experimental screen was performed by employing a full-factorial design consisting of five supersaturation levels, five temperature levels, and three central points, resulting in 28 experiments. The initial DoE plan for lamivudine (Table 2) was centered around a supersaturation of 2.4 and an isothermal temperature of 20 °C and for aspirin (Table 2), the DoE plan was centered around a supersaturation of 1.5 and a temperature of 20 °C.

**Table 2:** Adaptive Design of Experiment Plan for Optimization Iterations

	iteration			
	initial	1	2	3^a^
supersaturation	±0.4	±0.2	±0.1	±0.05
temperature	±10 °C	±10 °C	±5 °C	±2 °C

a If iterations continue past 3, then the same exploitative (small scope of experimental design space) plan is followed.

For the subsequent optimization, an objective function surface could not be fitted directly to the kinetic parameters measured experimentally for multiple reasons. First, as nucleation is a stochastic process dozens of experiments are required to sample the probability distribution of induction time values.^3^ Second, there is an inherent measurement uncertainty associated with the nucleation and growth rate parameters obtained via image analysis, as only a subset of sample particles are sampled. Consequently, multiple data points are required to reduce the uncertainties associated with these kinetic parameters.

As the aim of the optimization was to achieve convergence in as few experiments as possible, equations that describe the kinetics of crystallization were fit to the experimental data and used to smooth the surface of the objective. To smooth the surface of the objective function with domain knowledge, experimental data were plotted for each input parameter (temperature and supersaturation) with respect to each objective (induction time, nucleation rate, and growth rate) and eqs 1–5 (below)^30,31^ were fit to the data
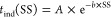
1
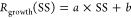
2
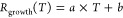
3
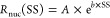
4
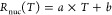
5where *t*_ind_ is the induction time, *R*_growth_ is the growth rate, *R*_nuc_ is the nucleation rate at a given supersaturation SS (where SS = 1 at equilibrium solubility), and temperature *T* and *a* and *b* are fitted parameter coefficients. The growth rate objective was weighted by a factor of 10, and this meant that all parameters sat within the same order of magnitude and thus on a comparable scale. There is no direct domain relationship between induction time and temperature, so this relationship was assumed as negligible, and therefore, parameters were not fitted. The parameters *A*, *a*, and *b* were obtained from the fitted equations, and the resulting fitted functions for induction time, nucleation rate, and growth rate were then mathematically manipulated^32^ so that the minimum of each function occurs at the objective target. The functions transformations used are given below with eq 6 being used to transform equations that feature exponential relationships between the objective and the input parameters (eqs 1 and 4), and eq 7 being used to transform equations that feature linear relationships (eqs 2, 3, and 5)
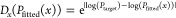
6
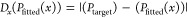
7where *P*_target_ is the target value for a given parameter, *P*_fitted_ is the fitted equation for a given parameter evaluated at the input value for either supersaturation, SS, or temperature, *T*, both here represented by *x*, and the difference between the target value *P*_target_ and fitted value *P*_fitted_(*x*) for a given target parameter is defined as the function *D*_*x*_[*P*_fitted_(*x*)]. The objective function for a given supersaturation and temperature, *f*(SS,*T*), could then be defined as follows
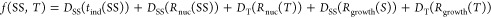
8

The minimum of the objective surface (within the parameter upper and lower bounds) was then calculated using multiple approaches: a genetic algorithm (GA), differential evolution (DE), covariance matrix adaptation evolution strategy (CMA-ES), and Nelder–Mead or pattern search approach. These algorithms were implemented in Python using the PyMOO^33^ library. The means of the values of supersaturation and temperatures corresponding to the predicted minimum from each algorithm were used as the center point for the next round of experiments. A smaller two-level full factorial DoE (Table 2) was then performed at this supersaturation and temperature. Following this round of experiments, the fitted functions were updated, the objective function surface was recalculated, and a new average minimum was predicted for the next round of experiments. This loop was repeated until the termination criteria were met. The subsequent DoE plans can be seen in Table 2. Using multiple algorithms to predict the next best experiment allowed us to remove outliers (see Tables S1 and S2).

#### 3.2.2 Optimization Approach 2: Bayesian Optimization

Three center points were taken as initial values and the difference between the target objectives and the experimental values for the parameters of interest were included in the objective function using the equation below
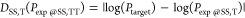
9where *P*_target_ is the target value for a given parameter, *P*_exp@SS,T_ is the experimental value for a given parameter at given supersaturation, SS, and temperature, *T*, inputs, and the difference between the log target value, *P*_target_, and log experimental value, *P*_exp@SS,T_, for a given supersaturation and temperature is defined as the function *D*_SS,T_(*P*_exp@SS,T_), where *P* is the parameter of interest. As stated earlier in the construction of eq 8, the relationship between induction and temperature can be assumed to be negligible; the same can be applied in eq 10 also.

The objective function assessed with kinetic parameters measured at a given supersaturation and temperature, *f*(SS,*T*), compared with the target kinetic parameters was then defined as follows
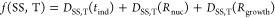
10

A Bayesian optimization algorithm was then implemented to determine the next experimental point to trial within the lower and upper bounds of the parameters. The algorithm was implemented in Python using the GPyOpt^34^ library using a Gaussian process probabilistic model and expected improvement acquisition function (see item 1 in the Supporting Information).

A Bayesian model with acquisition jitter (a scalar ratio between exploration: exploitation) of 0.001, 0.1, 1, and 10 as well as an adaptive dynamic model was performed. The criterion for changing the exploration/exploitation trade off was that if the objective function value falls below 10% of the maximum objective function value, the acquisition jitter is set to 1. Then, if the objective function value falls below 5% of the maximum, then the acquisition jitter is assigned a value of 0.1. In summary, this approach starts by selecting experiments across the whole design space and then adapts its search purpose to focus on finding the true optimum solution. This discrete-value adaptive approach makes sense for a crystallization given the potential for many local optima and that experiments are done in batch. A continuous-value adaptive approach to changing the acquisition jitter could be implemented in fast moving experiments such as flow chemistry. Similar to the DoE optimizations, the termination criteria for convergence were a change in temperature of less than 2 °C and a change in supersaturation of less than 0.02 between recommended experiments. Unlike the DoE optimizations, no domain knowledge in the form of physical equations that describe the system was needed as Gaussian processes can adapt the function used allowing good fit to data with many minia and large uncertainties.

## 4 Results and Discussion

Lamivudine recrystallizing from ethanol typically has relatively slow kinetics [i.e., large MSZW and induction times in the hours (SS ∼ 2)],^22^ whereas aspirin in ethyl acetate typically has fast kinetics [i.e., narrow MSZW and induction times in the minutes (SS ∼ 2)].^27^ Comparing the performance of the DoE and BO optimization methods for APIs with different inherent kinetic profiles can provide evidence for the generalizability of application of these methods.

### 4.1 Design of Experiment with Surface Minimization

Overall, the DoE approach allowed for sufficient data collection of measured kinetic parameters and, after applying domain knowledge, allowed for visualization of the data on a 3D surface. The minimum point of the surface (the only optimum value) was then found each time by search-based algorithms (refer to Section 3.2.1.).

The initial DoE screen consisting of 28 experiments investigated how supersaturation and temperature impacted each of the three objectives: induction time, nucleation rate, and growth rate. The minimum of the 3D objective function surface corresponded to a supersaturation of 2.88 and a temperature of 26.9 °C for lamivudine and a supersaturation of 1.21 and a temperature of 45.0 °C for aspirin. As discussed earlier, various approaches were used to identify the minimum, and the values determined by GA, DE, CM-AES, and pattern search consistently agreed (Tables S1 and S2). By contrast, the Nelder Mead values were discarded when calculating the mean value in early iterations, as this algorithm encountered calculation errors and only predicted the minimum under boundary conditions. The results over seven iterations of the optimization and experimental loop are shown in Table 3 where the median values of supersaturation and isothermal temperature predicted across all algorithms are presented.

**Table 3:** Predicted Optimum Supersaturations and Isothermal Experimental Temperatures for Lamivudine and Aspirin Over Multiple Iterations with the Objective Function Also Presented

iteration	lamivudine			aspirin		
	temperature (°C)	SS	obj. function value	temperature (°C)	SS	obj. function value
initial screen	26.9	2.88	0.86	45.0	1.21	1.00
2	26	2.63	0.81	50.0	1.18	0.90
3	24.9	2.56	0.73	22.62	1.12	0.84
4	22.4	2.28	0.72	19.13	1.14	0.72
5	22.97	2.31	0.68	12.97	1.14	0.68
6	23.73	2.36	0.61	5.98	1.16	0.57
7	24.12	2.36	0.61	5.0	1.16	0.57

**Figure 2:**
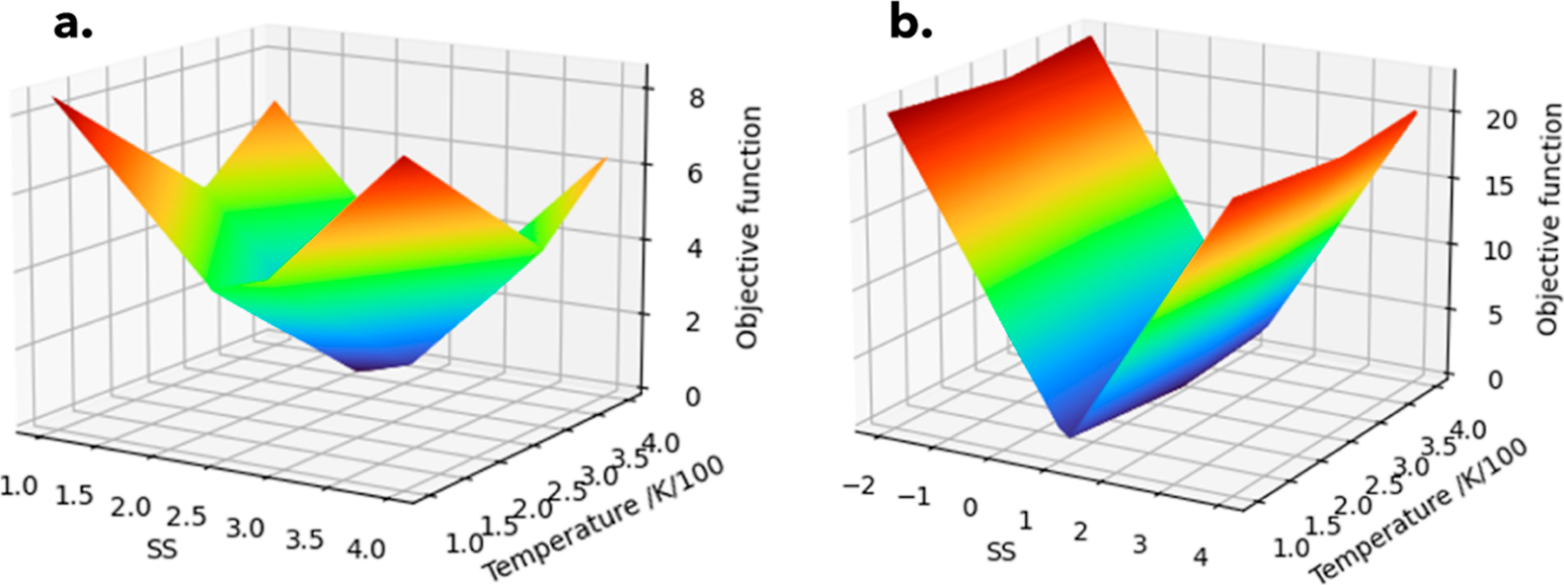
Surface plot of the objective function resulting from the adaptive DoE optimization experiments for lamivudine (a) and aspirin (b).

For both APIs, the termination criteria (see Section 3.2.1.) were achieved after the seventh iteration of the algorithm, equating to 70 experiments. These optimization approaches were consistent in predicting relatively similar values of supersaturation and temperature for the global minimum of the objective function for lamivudine. However, for aspirin, a series of supersaturation and temperature values resulted in the global minimum “valley” as seen in Figure 2.

The surface plot for aspirin has minimal dependence on temperature and is much more dependent on supersaturation such that small changes (±0.1) in the supersaturation had more impact on the crystallization kinetics than changes in the temperature. The surface plot for lamivudine shows a comparable impact on crystallization kinetics caused both by supersaturation and temperature.

DoE approaches are typically less suited to handle high levels of noise.^35^ The fitting of physical equations to smooth the objective function surface helped address this issue of noise^36^ associated with the stochastic nature of crystallization nucleation kinetics, and, arguably, significantly reduces the number of experiments required to generate a smooth enough surface to find a minimum. This in-part mechanistic model parameter fitting allowed for a second-order polynomial kinetic model to be defined for each API.

### 4.2 Bayesian Optimization

BO was applied using five different acquisition jitter level models to evaluate the performance from different exploration and exploitation weightings. These were as follows: highly exploitation-focused, exploitation-focused, exploration-focused, a model that balanced exploration versus exploitation, and an adaptive model which started as exploration-focused and moved to exploitation-focused as the objective function value decreased (see Section 3.2.2 for further details). The three center points of the domain space of interest (SS of 2.4 and temperature of 20 °C for lamivudine and SS of 1.5 and temperature of 20 °C for aspirin), and the related extracted kinetic data from the initial DoE plan, were used as initial values for the Bayesian model.

As expected, exploitation-focused models, i.e., acquisition jitter of 0.001 to 0.1, terminated quickly but still had high values (relative to other models) for the objective function for both lamivudine and aspirin, indicating that optimization likely found a local rather than global minimum (Table 4). The model which balanced the focus between exploitation and exploration, i.e., acquisition jitter of 1, terminated for both lamivudine and aspirin after six experiments again with high objective function values indicative of local minima (Table 4). The exploration-focused model, i.e., acquisition jitter of 10, achieved significantly lower objective function values for both APIs compared with the exploitation-focused model and the model that balanced exploration and exploitation models. However, the exploration-focused model terminated after only 31 experiments for lamivudine and 25 experiments for aspirin (Table 4).

**Table 4:** Predicted Optimum Supersaturations and Isothermal Experimental Temperatures for Lamivudine and Aspirin Across Different Exploration/Exploitation Bayesian Models

acquisition jitter model	lamivudine				aspirin			
	temperature (°C)	SS	obj. function value	no. of exp.	temperature (°C)	SS	obj. function value	no. of exp.
0.001	20	2.43	1.90	8	20	1.49	1.98	6
0.1	19.82	2.58	1.76	6	19.52	1.50	1.98	8
1	20.95	2.63	1.38	6	20	1.51	1.98	6
10	29.8	2.57	0.86	31	14.39	1.36	0.40	25
adaptive	17.9	2.86	0.40	32	18.17	1.27	0.40	15

To integrate the advantages of exploration-focused models (lower objective function values indicative of a global minimum) and exploitation-focused models (faster convergence), an adaptive Bayesian model was explored. This acquisition jitter for this model started high (10), i.e., exploration-focused, and was reduced stepwise with steps down in value triggered by reductions in objective function values until the termination criteria was met. In other words, an objective function value between 5 and 10% of the maximum value (this was 8% after 13 experiments for lamivudine and 6% after 14 experiments for aspirin) triggered the change of acquisition jitter from 10 of 1. Termination criteria were met for aspirin after 1 experiment at an acquisition jitter of 1. In the lamivudine optimization, a reduction in the objective function value to a value of 3% of the maximum triggered the acquisition jitter to change from 1 to 0.1 after a further 19 experiments. The results from the next experiment following this change met termination criteria. If the problem was more complex or the termination criteria more stringent; then the acquisition jitter could be further reduced; however, this was unnecessary here. In total termination criteria were satisfied after 32 experiments for lamivudine and 15 experiments for aspirin (Table 4). This difference in the number of experiments required is indicative of the differences between fast and slow kinetic regimes for the two APIs. Aspirin has a smaller “sweet spot” and a large gradient into the minimum due to the large impact on crystallization kinetics from small changes in process conditions particularly SS and as such the BO algorithm can find the optimum more efficiently.

**Figure 3:**
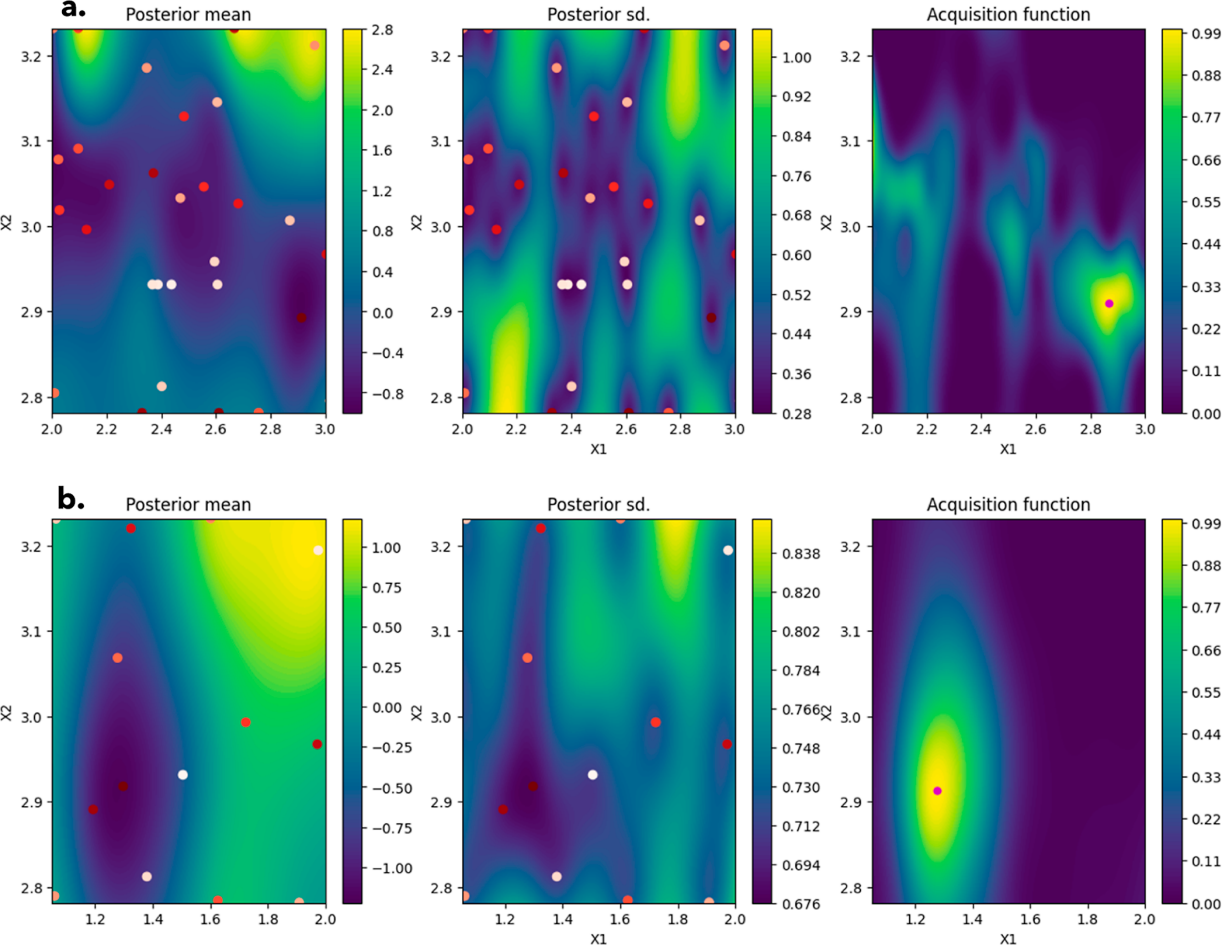
Plot of posterior mean, posterior standard deviation (sd), and acquisition function for lamivudine (a) and aspirin (b) for the final iteration of the adaptive exploration/exploitation model. The *X* and *Y* axes, labeled as *X*1 and *X*2, correspond to the supersaturation and temperature (respectively) input values at which the posterior mean, posterior standard, and acquisition function are evaluated.

Figure 3 shows the 2D heat maps for the objective function value (here labeled posterior mean referring to the mean of the posterior distribution^37^ output in BO), the uncertainty associated with these values (posterior standard deviation), and the acquisition function (an expected improvement algorithm used to weight the optimization toward exploration or exploitation). While some parts of the design space still have areas with higher levels of uncertainty (i.e., posterior standard deviation of ∼0.8 and above), these areas also correlate to points where the value of the objective function (i.e., posterior mean values) is also predicted to be high. Thus, these areas are unlikely to correspond to the global minimum, i.e., objective of the optimization.

### 4.3 Comparison of the Optimization Methods

It is worth focusing on the results from AdBO as this approach either performed as well as or better than other BO configurations tested and shows most promise as a general experimental optimization approach. As the objective functions for BO and DoE approaches investigate the absolute difference between the experimental or fitted outcome and the target values for each induction time, nucleation rate, and growth rate, we can compare the objective function values for both methods. All target parameters for induction time, nucleation rate, and growth rate were satisfied for both lamivudine and aspirin with experimental validation (Figure S6—with reference to reliability of each measurement), and XRPD (Figures S7 and S8) was run on each crystal sample recovered to confirm the target polymorphic form for both lamivudine and aspirin was produced.

**Figure 4:**
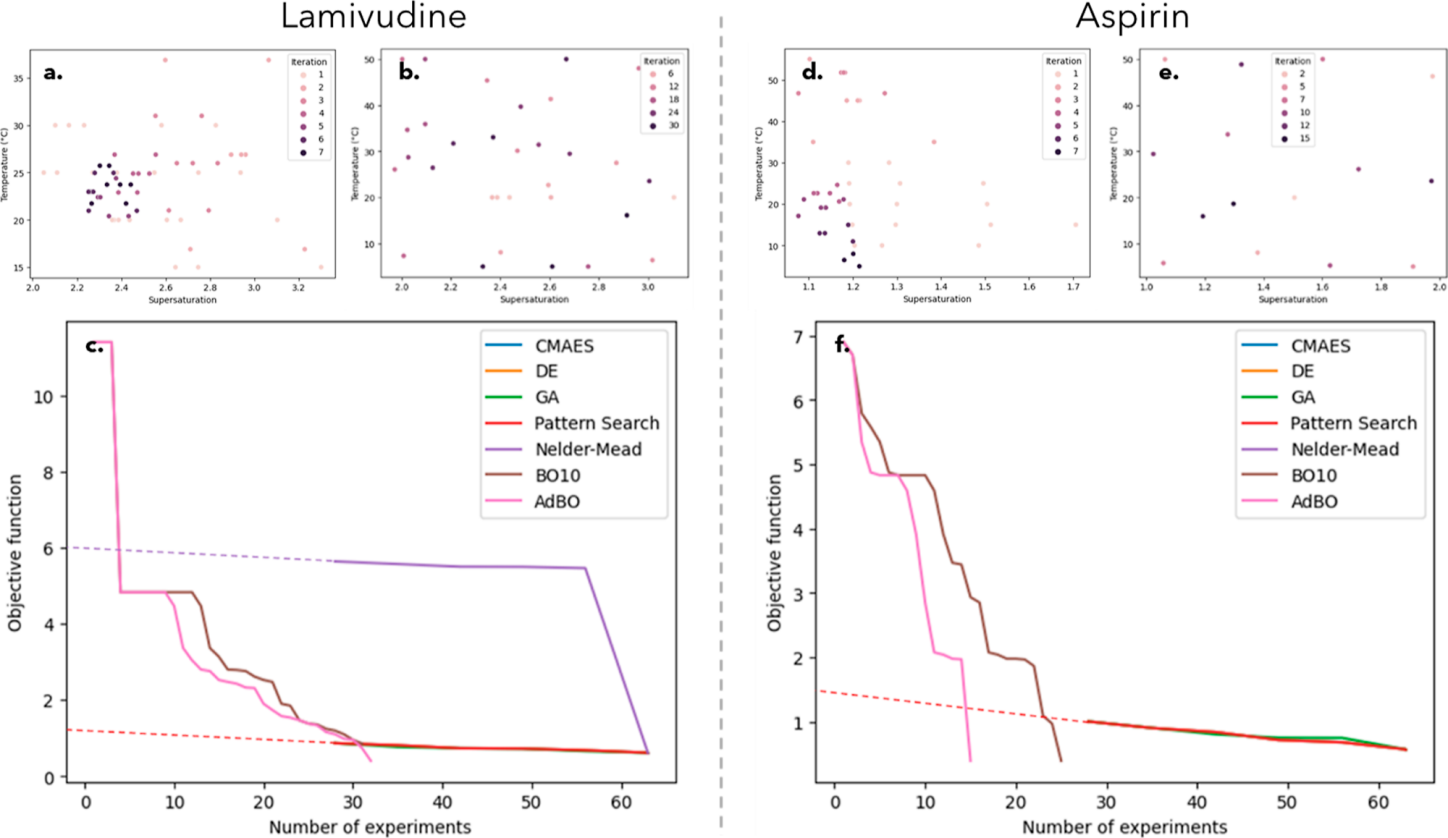
Comparison between different optimization approaches, DoE for lamivudine (a) and aspirin (d) and AdBO for lamivudine (b*) and aspirin (e*) and algorithm performance in reducing the objective function for process condition optimization for small-scale batch cooling crystallization of lamivudine (c) and aspirin (f). The dashed lined represents an extrapolation back to experiment 0, as the DoE methods required a 28 initial screening experimental plan.*The numbering on the legends for these figures were not sequential as the AdBO method was a 1 experiment/iteration approach and thus would make the legend too large to display.

The BO methodology for optimizing kinetic parameters required fewer experiments when compared with the DoE approach (Figure 4). Specifically, AdBO optimization of lamivudine crystallization used 50% less material and required 54% less experiments than DoE methodology. Further improvements were seen for aspirin crystallization where AdBO used 75% less material and required 79% less experiments than the DoE optimization. This improvement in experimental efficiency is predominantly due to the fact that the AdBO method does not require an initial screen of experiments.

As shown in Figure 4c,f, the AdBO method reaches the lowest objective function value and satisfies the termination criteria in the least number of experiments, suggesting that the AdBO method can outperform both the fixed acquisition jitter Bayesian models and the DoE method. While the DoE method achieved a relatively low objective function through initial screening, the AdBO method continues to drive the trends in objective function even lower. Notably, the AdBO model demonstrates a significant improvement over the BO10 (Bayesian with an acquisition jitter of 10) for aspirin, achieving a low objective function in 10 fewer experiments. Furthermore, AdBO’s faster reduction of the objective function and satisfaction of achieving the termination criteria comes at no additional computational cost as all the algorithms employed in this study exhibited comparable average execution times (from 3 to 4 s), with no statistically significant differences observed (Figure S5).

The inherent noise in the objective function also presented no noticeable challenge for the AdBO optimization. The AdBO method, as applied, was “blind” to the physics of the experiment in that no domain knowledge was required to achieve a fit for the objective function. This lack of reliance on equations that describe the physics of the system provides us with a potentially generalizable method that can handle the inherent stochastic nature of induction time and the higher levels of noise associated with fewer numbers of experimental points.^38^ These results also suggest that the AdBO method may have potential application to optimize other parameters for which the physics of the system is complex or poorly understood as well as other physical processes beyond crystallization.

Both approaches (DoE and AdBO) discussed in this paper have also shown significant improvements over a grid search, with Bayesian methods requiring the least experimental work. A grid search of the design space for lamivudine and aspirin would require 1125 and 1069 experiments, respectively, based on the increments of the termination criteria and the span of the design space for each variable for each API.

## 5 Conclusions

This study demonstrated the successful application of two optimization methods for crystallization kinetic parameters of two APIs with the approaches implemented in the Python libraries, PyMOO and GPyOpt. By minimizing the total sum of the differences between target and experimental values, these algorithms have successfully achieved the desired target values for induction time, nucleation rate, and growth rate that relate to attainable conditions for a viable industrial process design. Two case studies of lamivudine and aspirin were explored to assess the effectiveness of the algorithms for APIs that display widely differing crystallization kinetics. The DoE and AdBO methods identified the Pareto optimal process conditions, specifically supersaturation and temperature, essential for optimizing crystallization processes. Notably, both methods yielded low objective function values (0.61 for DoE lamivudine and 0.57 for DoE aspirin and 0.40 for AdBO both lamivudine and aspirin with respect to initial values upward of 11 for lamivudine and 7 for aspirin). The savings, in terms of time and material, of using AdBO over DoE methods can be estimated as between a 15–80 kWh reduction in energy and specifically £20,000/kg for lamivudine and £60/kg for aspirin.

The effective application of the AdBO method to these two APIs is promising; however, further study across a wider range of APIs is required to confirm the generalizability of this approach to the wide range of physicochemical properties presented by new pharmaceutical molecules. It will be important to address known limitations of the method, such as increasing the dimensionality of this problem beyond 20 inputs/outputs (e.g., stirring rate, rate of antisolvent addition, heating/cooling rate, and morphology) could reduce the algorithmic performance.^39,40^ The incorporation of these more complex parameters will have a complex relationship with nucleation rate and growth rate, and as such convergence of the algorithm may require further experimentation or an initial training data set to achieve high algorithmic performance. Furthermore, in this work, we relied on prior knowledge of the MSZW in specific solvents system tested when constraining the design space search. However, the approach can be phased to explore potential process conditions to evaluate MSZW under different conditions and then feed into more detailed kinetic parameter studies.

Even with these limitations, this study clearly shows that using BO to guide experimental design allows for a faster, targeted, and more sustainable approach to API crystallization kinetics data collection in pharmaceutical development. Furthermore, the AdBO model could transform automated crystallization data collection to autonomous experimental design enabling smart experimentation for crystallization kinetics to further enhance R&D productivity and process understanding. This implementation of BO has the potential to translate to meaningful cost savings for materials, resource, energy utilization, and chemical waste and to accelerate development timelines. This method could also be expanded to investigate the optimization of other numerical parameter objectives such as solute concentration, aspect ratio, and yield and applied to optimization of drug substant filtration^41^ and flow.^42^

## Supporting Information Available

AdBO code, DoE iterative results, Bayesian optimization figures, computational time analysis, experimental validation of optimized experiment, and XRPD patterns (PDF)
