# Supplementary material for: Comparative Study on Adaptive Bayesian Optimization for Batch Cooling Crystallization for Slow and Fast Kinetic Regimes
Source: Cryst Growth Des. 2024 Jan 29;24(3):1245–53. doi: 10.1021/acs.cgd.3c01225 (PMC10853904; doi:10.1021/acs.cgd.3c01225)
Supplement: Supplementary file 1 — cg3c01225_si_001.pdf [file cg3c01225_si_001.pdf]

Comparative Study on Adaptive Bayesian Optimization for Batch Cooling Crystallization for Slow and Fast Kinetic Regimes

Thomas Pickles[a], Chantal Mustoe[a], Cameron J. Brown[a], Alastair J. Florence[a*]

[a]CMAC Future Manufacturing Research Hub, Technology and Innovation Centre, The University of Strathclyde, Glasgow, G1 1RD, United Kingdom

***alastair.florence@strath.ac.uk**

1. ESI

```
def f(x,y):
    return x**2 + x + y**2 + y
GPyOpt.methods.BayesianOptimization(f = f,
                                    domain=bounds,
                                    model_type='GP',
                                    acquisition_type ='EI',
                                    acquisition_jitter = 0.001 to 10,
                                    X=xy_init,
                                    Y=z_init)
```

Item 1. Bayesian optimisation code implemented in GPyOpt – using a simple 2D parabola function to aim for minimums on the surfaces of the initial data, domains set out as the input parameter bounds, a Gausian process probabilistic model, an expected improvement acquisition function, a varied exploration/ exploitation acquisition jitter and experimental data inputs of X (supersaturation (x) and temperature (y)) and the measured inputs of Y (crystallisation kinetics (z)).

Table 1. Individual performance for next best experiment from each algorithm for DoE (PyMOO) for lamivudine.

Iteration	Algorithm	Results
**Initial Screen**	GA	Supersaturation - 2.88<br>Temperature - 27.06 °C
	DE	Supersaturation - 2.88<br>Temperature - 26.92 °C
	CMAES	Supersaturation - 2.88<br>Temperature - 26.92 °C
	Nelder Mead	Supersaturation - 2.88<br>Temperature - -273.15 °C
	Pattern Search	Supersaturation - 2.88<br>Temperature - 26.92 °C
**Supersaturation of 2.88 +/- 0.2 and temperature of 26.9 +/- 10 °C will be used.**		
2	GA	Supersaturation - 2.63<br>Temperature - 25.65 °C

	DE	Supersaturation - 2.63<br>Temperature - 25.97 °C
	CMAES	Supersaturation - 2.63<br>Temperature - 25.97 °C
	Nelder Mead	Supersaturation - 2.62<br>Temperature - -273.15 °C
	Pattern Search	Supersaturation - 2.63<br>Temperature - 25.97 °C
**Supersaturation of 2.63 +/- 0.1 and temperature of 26.0 +/- 5 °C will be used.**		
3	GA	Supersaturation - 2.51<br>Temperature - 24.46 °C
	DE	Supersaturation - 2.56<br>Temperature - 24.87 °C
	CMAES	Supersaturation - 2.56<br>Temperature - 24.87 °C
	Nelder Mead	Supersaturation - 2.56<br>Temperature - -273.15 °C
	Pattern Search	Supersaturation - 2.56<br>Temperature - 24.87 °C
**Supersaturation of 2.56 +/- 0.05 and temperature of 24.9 +/- 2 °C will be used.**		
4	GA	Supersaturation - 2.3<br>Temperature - 22.18 °C
	DE	Supersaturation - 2.28<br>Temperature - 22.4 °C
	CMAES	Supersaturation - 2.28<br>Temperature - 22.4 °C
	Nelder Mead	Supersaturation - 2.28<br>Temperature - -273.15 °C
	Pattern Search	Supersaturation - 2.28<br>Temperature - 22.4 °C
**Supersaturation of 2.28 +/- 0.05 and temperature of 22.4 +/- 2 °C will be used.**		
5	GA	Supersaturation - 2.31<br>Temperature - 22.97 °C
	DE	Supersaturation - 2.31<br>Temperature - 22.97 °C
	CMAES	Supersaturation - 2.31<br>Temperature - 22.97 °C
	Nelder Mead	Supersaturation - 2.31<br>Temperature - -273.15 °C
	Pattern Search	Supersaturation - 2.31<br>Temperature - 22.97 °C
**Supersaturation of 2.31 +/- 0.05 and temperature of 22.97 +/- 2 °C will be used.**		
6	GA	Supersaturation - 2.36<br>Temperature - 23.73 °C

	DE	Supersaturation - 2.36<br>Temperature - 23.73 °C
	CMAES	Supersaturation - 2.36<br>Temperature - 23.73 °C
	Nelder Mead	Supersaturation - 2.36<br>Temperature - 23.73 °C
	Pattern Search	Supersaturation - 2.36<br>Temperature - 23.73 °C
**Supersaturation of 2.36 +/- 0.05 and temperature of 23.73 +/- 2 °C will be used.**		
7	GA	Supersaturation - 2.36<br>Temperature - 24.12 °C
	DE	Supersaturation - 2.36<br>Temperature - 24.12 °C
	CMAES	Supersaturation - 2.36<br>Temperature - 24.12 °C
	Nelder Mead	Supersaturation - 2.36<br>Temperature - 24.11 °C
	Pattern Search	Supersaturation - 2.36<br>Temperature - 24.12 °C
**Termination criteria of +/- 2 °C & 0.02 SS has been met**		

Table 2. Individual performance for next best experiment from each algorithm for DoE (PyMOO) for aspirin.

Iteration	Algorithm	Results
**Initial Screen**	GA	Supersaturation - 1.21<br>Temperature - 45.16 °C
	DE	Supersaturation - 1.21<br>Temperature - 45.04 °C
	CMAES	Supersaturation - 1.21<br>Temperature - 45.04 °C
	Nelder Mead	Supersaturation - ERROR<br>Temperature - ERROR
	Pattern Search	Supersaturation - 1.21<br>Temperature - 45.04 °C
**Supersaturation of 1.21 +/- 0.2 and temperature of 45 +/- 10 °C will be used.<br>Minimum SS to be trialled is 1.11.**		
2	GA	Supersaturation - 1.18<br>Temperature - 52.06 °C
	DE	Supersaturation - 1.18<br>Temperature - 51.75 °C

	CMAES	Supersaturation - 1.18<br>Temperature - 51.75 °C
	Nelder Mead	Supersaturation - ERROR<br>Temperature - ERROR
	Pattern Search	Supersaturation - 1.18<br>Temperature - 51.75 °C
**Supersaturation of 1.18 +/- 0.1 and temperature of 51.75 +/- 5 °C will be used. (Incorrect DoE plan was used here)**		
3	GA	Supersaturation - 1.12<br>Temperature - 22.62 °C
	DE	Supersaturation - 1.11<br>Temperature - 22.62 °C
	CMAES	Supersaturation - 1.11<br>Temperature - 22.62 °C
	Nelder Mead	Supersaturation - ERROR<br>Temperature - ERROR
	Pattern Search	Supersaturation - 1.11<br>Temperature - 22.62 °C
**Supersaturation of 1.12 +/- 0.05 and temperature of 22.62 +/- 2 °C will be used.**		
4	GA	Supersaturation - 1.13<br>Temperature - 19.13 °C
	DE	Supersaturation - 1.14<br>Temperature - 19.13 °C
	CMAES	Supersaturation - 1.14<br>Temperature - 19.13 °C
	Nelder Mead	Supersaturation - ERROR<br>Temperature - ERROR
	Pattern Search	Supersaturation - 1.14<br>Temperature - 19.13 °C
**Supersaturation of 1.14 +/- 0.05 and temperature of 19.13 +/- 2 °C will be used.**		
5	GA	Supersaturation - 1.12<br>Temperature - 12.94 °C
	DE	Supersaturation - 1.14<br>Temperature - 12.97 °C
	CMAES	Supersaturation - 1.14<br>Temperature - 12.97 °C

	Nelder Mead	Supersaturation - ERROR<br>Temperature - ERROR
	Pattern Search	Supersaturation - 1.14<br>Temperature - 12.97 °C
**Supersaturation of 1.14 +/- 0.05 and temperature of 12.97 +/- 2 °C will be used.**		
6	GA	Supersaturation - 1.16<br>Temperature - 5.98 °C
	DE	Supersaturation - 1.16<br>Temperature - 5.98 °C
	CMAES	Supersaturation - 1.16<br>Temperature - 5.98 °C
	Nelder Mead	Supersaturation - 1.16<br>Temperature - 5.98 °C
	Pattern Search	Supersaturation - 1.16<br>Temperature - 5.98 °C
**Supersaturation of 1.16 +/- 0.05 and temperature of 5.98 +/- 2 °C will be used.**		
7	GA	Supersaturation - 1.16<br>Temperature - 5 °C
	DE	Supersaturation - 1.16<br>Temperature - 5 °C
	CMAES	Supersaturation - 1.16<br>Temperature - 5 °C
	Nelder Mead	Supersaturation - 1.16<br>Temperature - 5 °C
	Pattern Search	Supersaturation - 1.16<br>Temperature - 5 °C
**Termination criteria of +/- 2 °C & 0.02 SS has been met**		

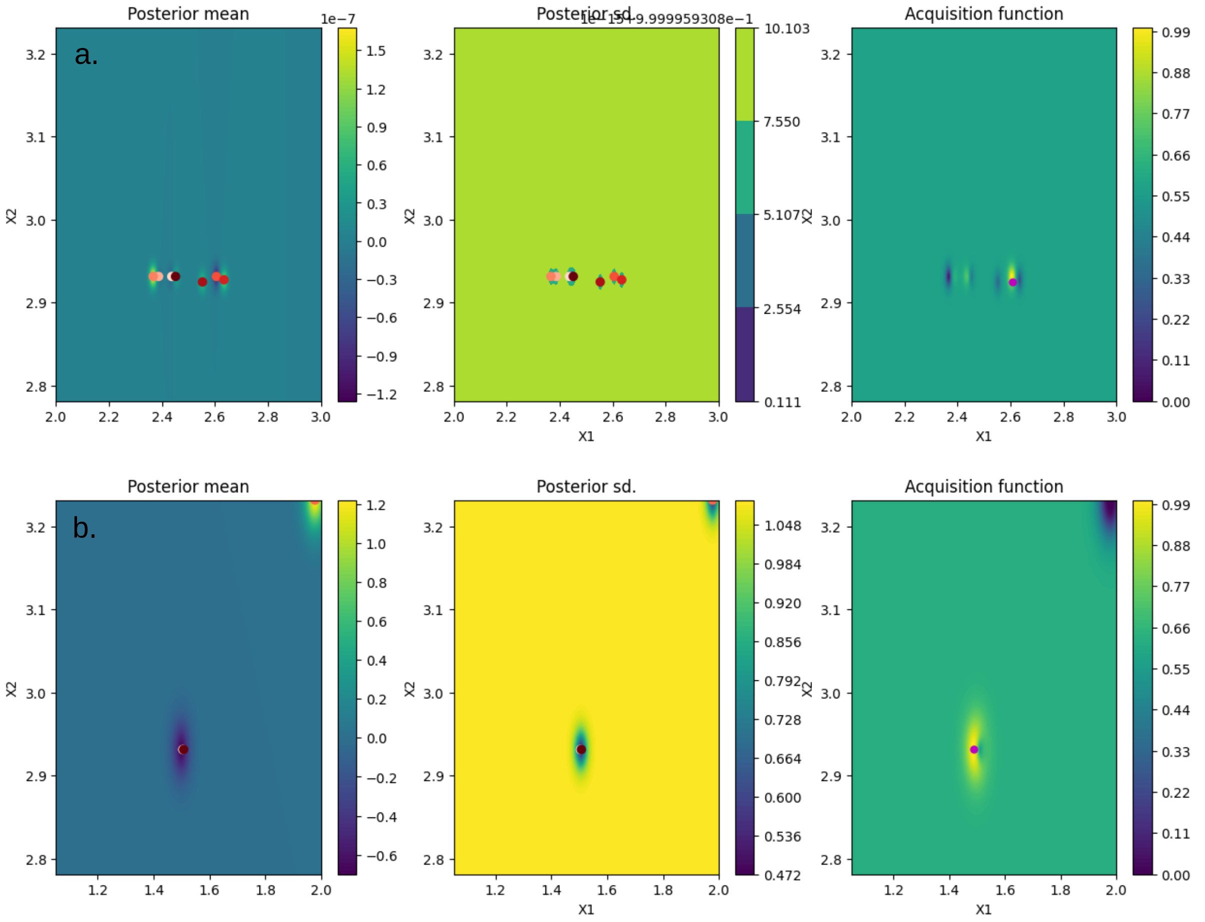

Figure 1. Plot of posterior mean, posterior standard deviation, and acquisition function for lamivudine (a) and aspirin (b) for the final iteration of the acquisition jitter = 0.001.

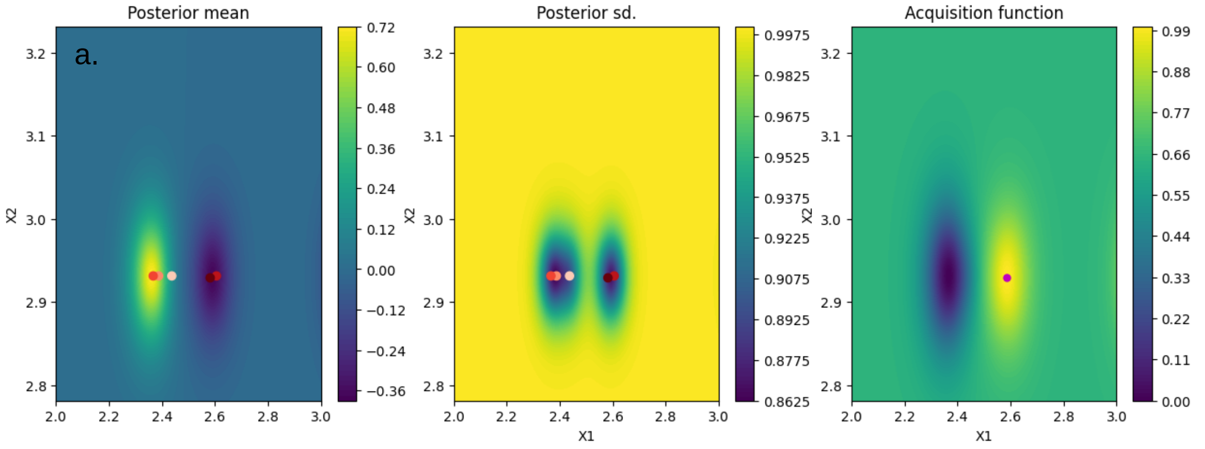

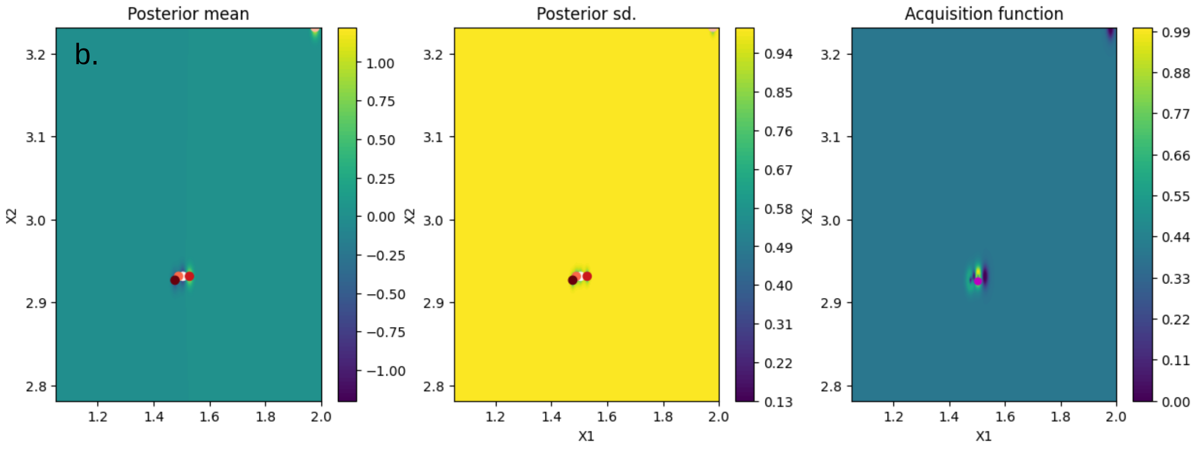

Figure 2. Plot of posterior mean, posterior standard deviation, and acquisition function for lamivudine (a) and aspirin (b) for the final iteration of the acquisition jitter = 0.1.

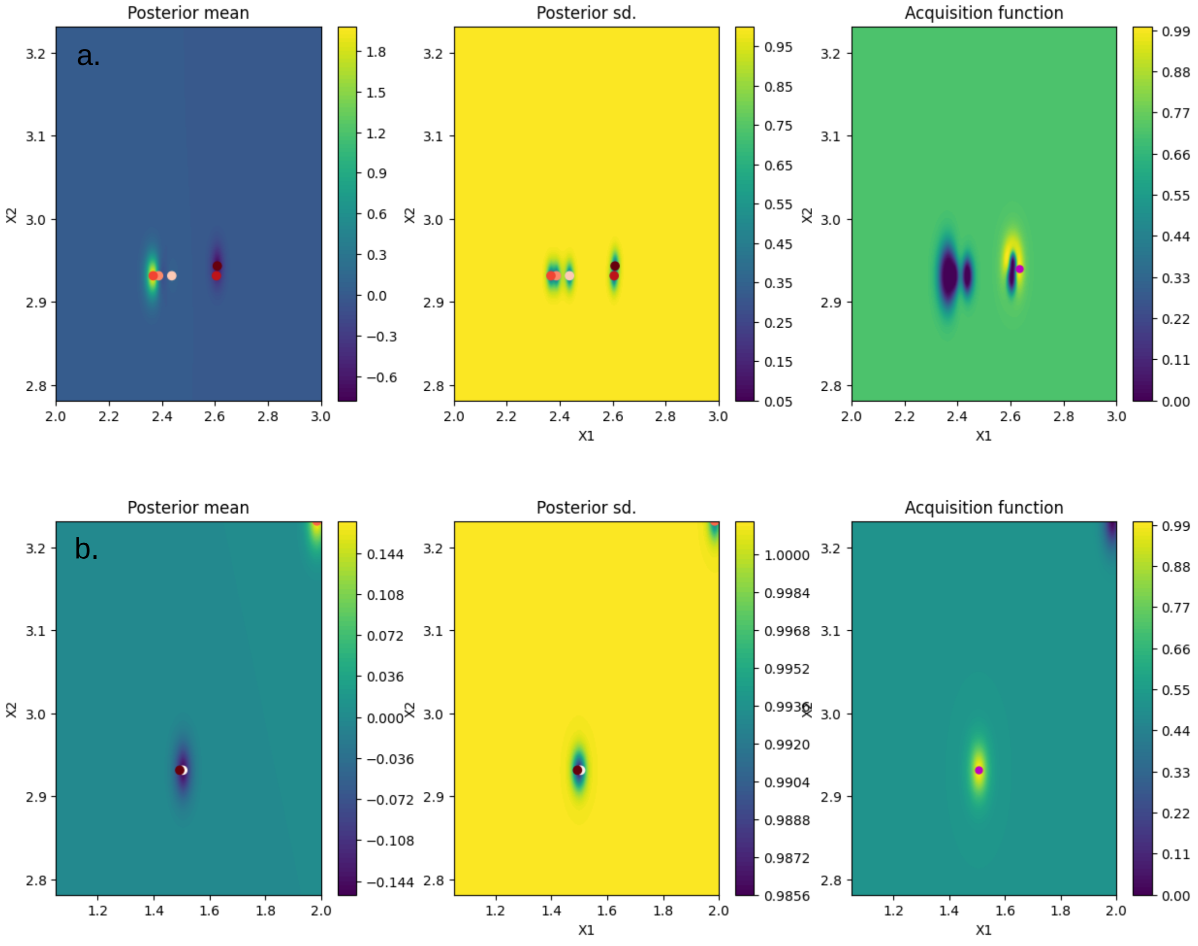

Figure 3. Plot of posterior mean, posterior standard deviation, and acquisition function for lamivudine (a) and aspirin (b) for the final iteration of the acquisition jitter = 1.

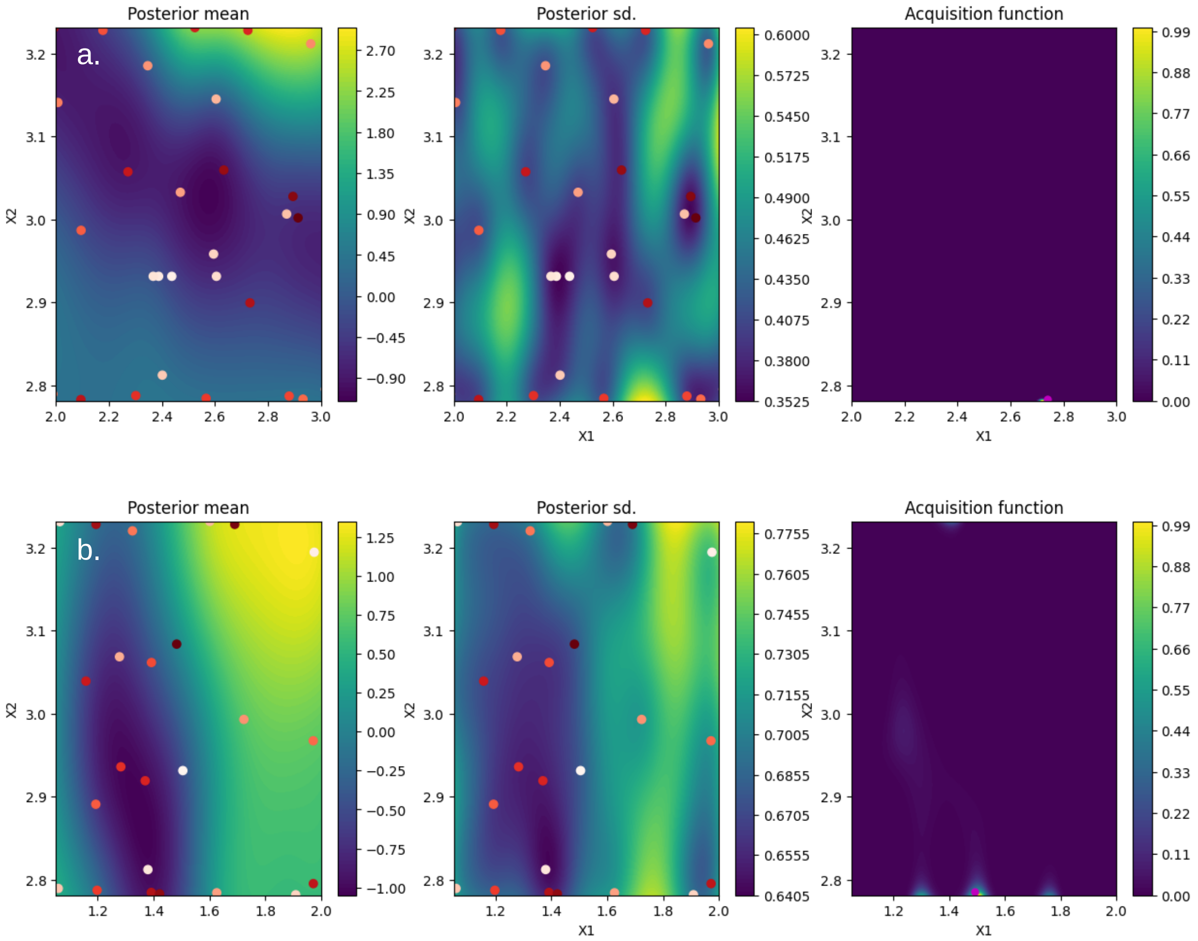

Figure 4. Plot of posterior mean, posterior standard deviation, and acquisition function for lamivudine (a) and aspirin (b) for the final iteration of the acquisition jitter = 10.

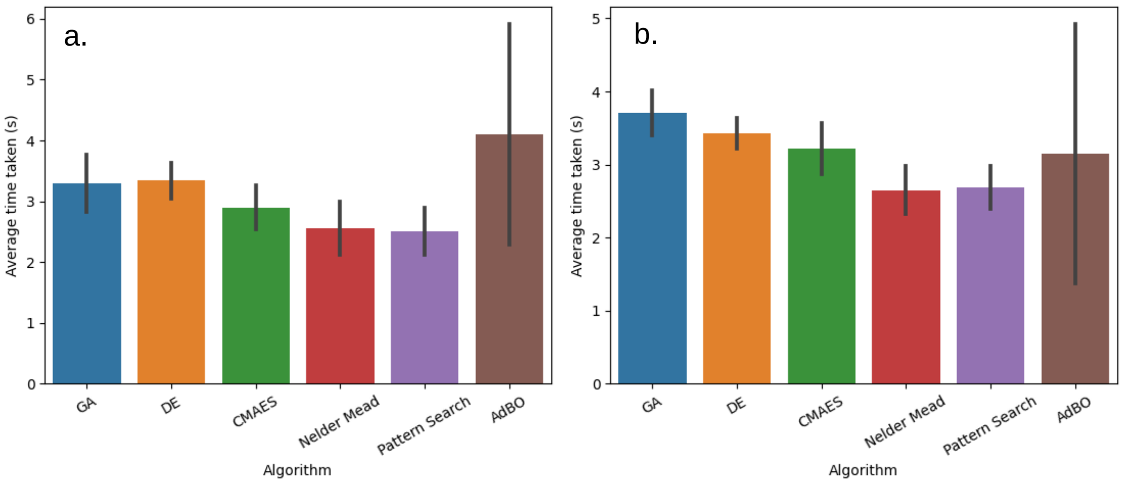

Figure 5. Computational time required to perform each optimisation algorithm, averaged over every iteration, for lamivudine (a) and aspirin (b). There is a general trend that Nelder Mead and Pattern Search performed the fastest but there are no statistically significant differences. Additionally, as all algorithm times were below an average of 5 s it can be deemed that due to the large time expense associated to the practical crystallisation experiment that algorithm time is trivial in the whole process loop.

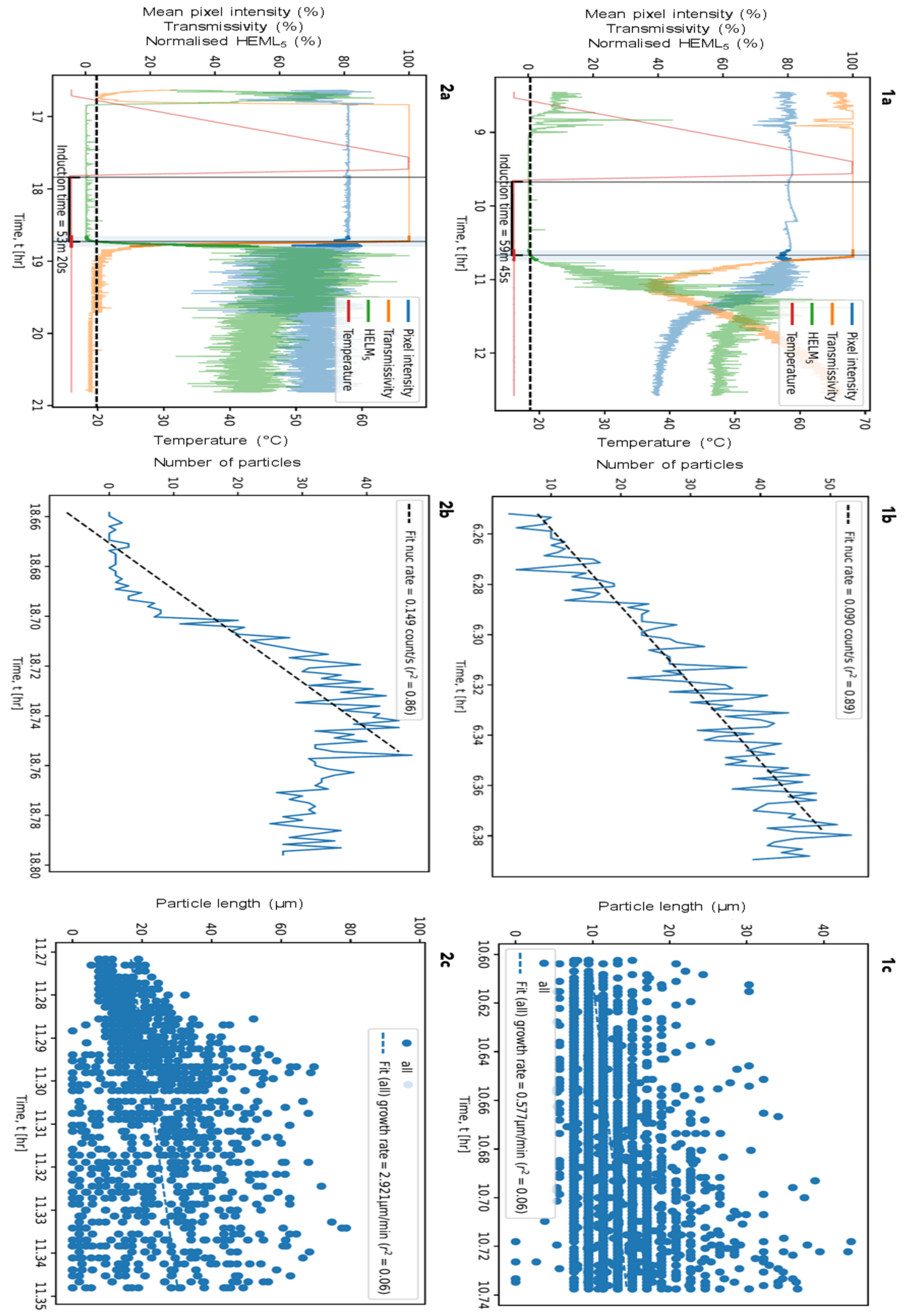

Figure 6. Induction time plot (1a), nucleation rate plot (1b), and growth rate plot fitted to all bins (1c) for optimised run of lamivudine and induction time plot (2a), nucleation rate plot (2b), and growth rate plot fitted to all bins (2c) for optimised run of aspirin. The low R^2 for the growth rate graphs is due to fitting the growth rate to all data rather than a particular bin size to increase population size. The use of 5 experiment cycles allowed for increased parameter reliability for the stochastic nature of induction time and the noisy fitting of growth rates.

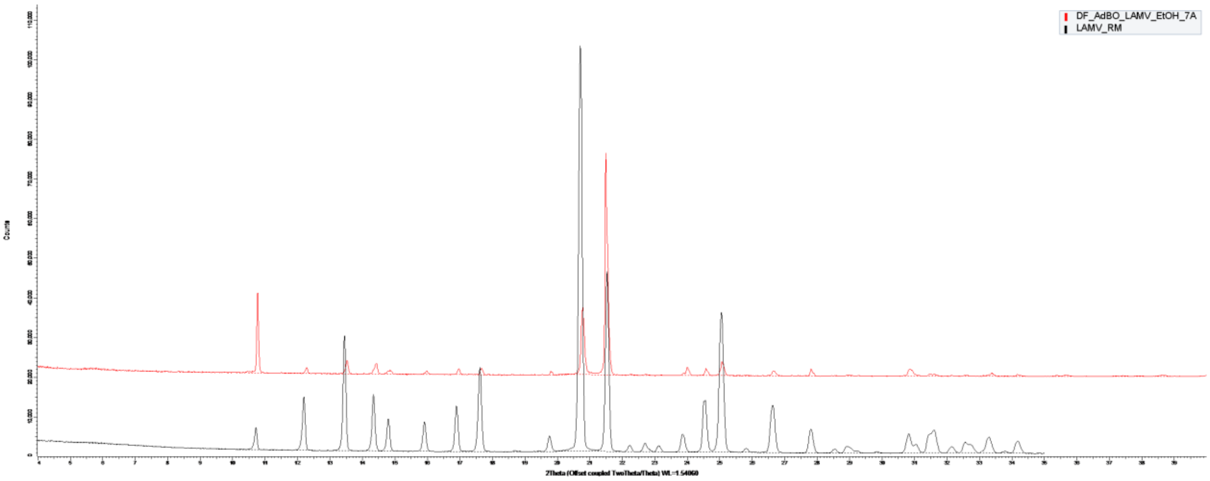

Figure 7. XRPD pattern for recrystallised lamivudine overlayed against lamivudine raw material.

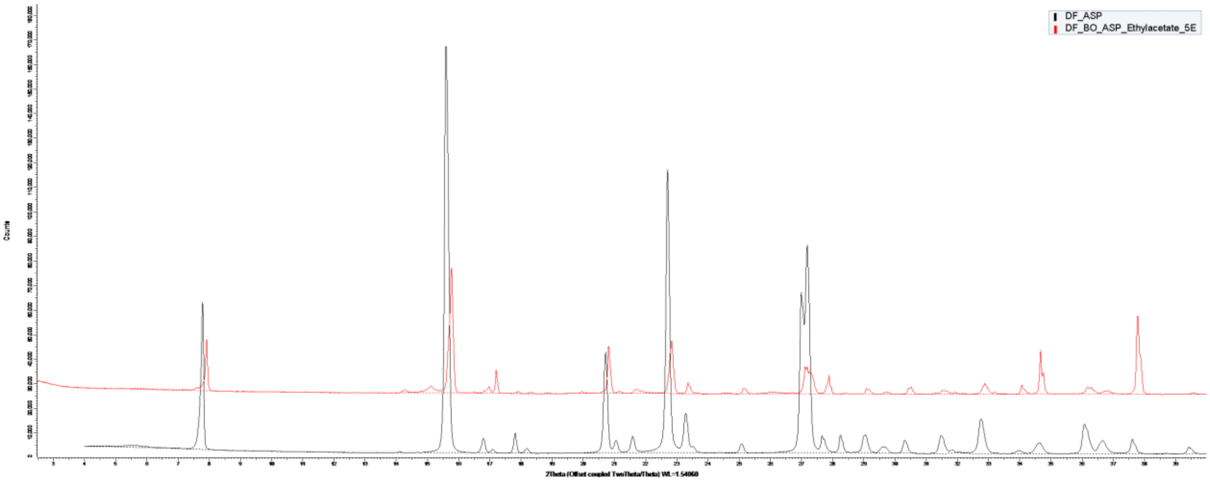

Figure 8. XRPD pattern for recrystallised aspirin overlayed against aspirin raw material.
